# Tissue print of prostate biopsy: a novel tool in the diagnostic procedure of prostate cancer

**DOI:** 10.1186/1746-1596-6-34

**Published:** 2011-04-13

**Authors:** Adriano Angelucci, Gianna Pace, Patrizia Sanità, Carlo Vicentini, Mauro Bologna

**Affiliations:** 1 Department of Experimental Medicine, University of L'Aquila, L'Aquila, 67100, Italy; 2 Department of Health Sciences, University of L'Aquila, L'Aquila, 67100, Italy

## Abstract

**Background:**

Nowadays, the histological examination of prostate core needle biopsies is still regarded as the gold standard in the diagnosis of prostate cancer (PCa). We investigated if the tissue print of core needle biopsy (biopsy print) could be used as adjunctive molecular investigative procedures in conjunction with routine histological examination of biopsy to improve PCa diagnosis.

**Methods:**

The direct contact of PCa core biopsy to nitrocellulose membrane resulted in the release of a cellular micropeel that was used for downstream analytical procedures.

**Results:**

By zymogram print-phoresis we demonstrated that matrix metalloproteases MMP-2 and MMP-9 could be visualized in biopsy prints and that the gelatinolytic activity was positively correlated with immunohistochemistry analysis of the same markers in matched bioptic specimens. Moreover, we compared the ability to detect the PCa-associated hypermethylation of GSTP1 promoter in DNA extracted from biopsy prints with those of the corresponding core needle biopsies. Biopsy prints demonstrated the same specificity of biopsies in detecting PCa (50%) while the sensitivity and the positive predictive value were lower than biopsies (56% vs 78% and 63% vs 70%, respectively).

**Conclusions:**

Biopsy print, combining a molecular point of view to the routinely hystopathological analysis of prostate biopsies, should be a useful tool to improve the diagnosis of PCa.

## Introduction

To date, the diagnosis of prostate cancer (PCa) is based upon the histological examination of prostate core needle biopsies. Other diagnostic procedures, although useful in improving the detection of PCa, such as measure of serum prostate specific antigen (PSA), still lack in adequate specificity to be used alone as diagnostic technique. The importance of prostate needle biopsies is supported by the continuous progress in biopsy scheme in the attempt to improve the detection rate of PCa. In fact, in the last few years the standard sextant biopsy has been replaced by 12 cores and even more extensive biopsy schemes [1,2]. Despite the current debate on the optimal biopsy scheme, the core needle biopsy is still regarded by urologists as the gold standard in the diagnosis of PCa. Moreover, in the attempt of better detecting PCa, several immunohistochemistry innovations have been developed, and some markers, as metalloproteases, has been associated to the progression of PCa [3].

The Gleason score offers a good description of the aggressiveness of the tumor, but it is insufficient in estimating the prognosis. In order to achieve an optimal therapeutic approach a molecular analysis of needle biopsy should be useful. However, a single core biopsy, due to its small size, cannot be reserved for other analytical procedures than pathologic examination. Although the idea of reproducing an exact anatomical copy of a tissue on a solid support is not a novelty, it has been applied mainly in botanic studies [4]. Tissue print is based on the transfer, by direct contact, of the superficial cellular contents of a fresh tissue to an adhesive or adsorptive surface. Tissue print of vegetal tissues on nylon or nitrocellulose membrane has demonstrated to be an effective tool to spatially detect the presence of specific proteins and mRNA [5]. Tissue copies on adhesive surface from rat and mouse tissues have been utilized in DNA-DNA and RNA-RNA hybridization assays and in immunochemistry [6-8]. In more recent studies, Gaston and colleagues have successfully applied the tissue print technique to the molecular investigation of PCa biopsy and of the entire prostate surface. They demonstrated that the cellular micropeel retained on the membrane is enough to detect proteins and mRNA in a wide range of application, from print-phoresis to RT-PCR [9].

Our aim has been to verify whether the biopsy print should be an adequate copy of the prostate biopsy and if it may be useful in the routinely clinical diagnostic investigation as based on an easy procedure and on a wide range of methods available for its analysis.

## Materials and methods

### Patients

We selected 18 consecutive patients with a histological diagnosis of PCa, who underwent radical retropubic prostatectomy (RRP) in our Urology Department of the G. Mazzini Hospital, Teramo, Italy. Patients were eligible if they did not undergo previous anti-hormonal, radiation or chemotherapies. We considered the serum PSA value at the diagnosis and Gleason score obtained after RRP. As controls we enrolled 6 patients affected by Benign Prostatic Hyperplasia (BPH) and diagnosed by the histopathological analysis of the tissue obtained after a transvescical retropubic adenomectomy (TV adenomectomy) or a transurethral resection prostatectomy (TURP). Our institutional review board approved the protocol. All patients signed the informed consent.

### Reagents and plasticware

Reagents, if not differently indicated, were purchased from Sigma-Aldrich (St. Luois, MI, USA). Plasticware was purchased by BD Biosciences (Franklin Lakes, NJ USA). Human prostate cancer cell line, PC3 was originally obtained by ATCC (Rockville, MD, USA) and maintained in DMEM supplemented with 10% fetal bovine serum (FBS), glutamine, and penicillin-streptomycin. Conditioned medium was obtained culturing PC3 cells for 48 h in FBS-free medium.

### Biopsy and tissue print

Three needle biopsies were performed immediately after the surgical treatment in the area where the tumor was localized. All the procedures were performed in aseptic atmosphere. Immediately, all three bioptic tissues were completely rinsed in phosphate buffer without Ca2+ and Mg2+ and then the excess of liquid was dried off by an absorbent paper. Each of the three biopsies were spread on a dry nitrocellulose membrane (0.2 μm Protran, Whatman plc, Kent, UK) and left to adhere for about 30 seconds. Then were delicately recovered with tweezers pulling the tissue from one end. The exceeding parts, without the print, were removed from nitrocellulose membranes. The resulting biopsy prints were conserved in a dry box at -20°C until processed.

### Zymogram print-phoresis

Gelatin zymogram was performed according to standard procedure with few modifications. A 10% SDS-polyacrylamide gel copolymerized with 0.1 mg/ml gelatine was utilized placing tissue print on the top of the resolving gel and then pouring the 6% SDS-polyacrylamide stacking gel. At the end of the run gel was washed three times with 50 mM Tris-HCl (pH 7.4) containing 2% Triton X-100 for 15 minutes under agitation to remove SDS, and was incubated with Tris-HCl (pH 7.4) plus 10 mM CaCl_2 _and 200 mM NaCl for 24 hours at 37°C. Gel was then fixed and stained with 0.1% Coomassie Blue solution. Enzyme digested regions were identified as white bands against a blue background.

### Immunohistochemistry

*The three *core needle biopsies were fixed in 4% formaldehyde in 0.1 M phosphate buffer, pH 7.2, and embedded in paraffin. Slide-mounted tissue sections were incubated with the anti-MMP2 or anti-MMP9 primary antibodies (Santa Cruz, CA, USA) for 1 h at room temperature. Antibody binding was revealed using the Ultra-Vision detection system anti-Polyvalent HRP/diaminobenzidine kit according to the manufacturer's instructions. Each case was evaluated blindly by two independent readers (A. A. and P. G.). The number of cells expressing the marker was assessed using a semiquantitative three-grade scale (score 0 = 0%, score 1 = 0-50%, or score 2 >50%). Cases in which the two observers had obtained different results were collectively reviewed, and a consensus was obtained.

### Methylation-specific PCR

*The three *needle biopsies obtained by focusing on the area of the tumor and the related tissue prints were incubated with lysis buffer (10 mmol/L Tris-HCl, pH 8.0, 0.1 mmol/L EDTA, 1% v/v SDS) containing 2 mg/ml proteinase K at 55°C for 4 h. DNA was then extracted with 1:1 v/v phenol/chloroform and precipitated with 50 mmol/L sodium acetate in ethanol. The concentration of extracted DNA was measured with spectrometry, and a range of 50-150 ng DNA was used in the following steps. Using the EpiTec Bisulfite Kit (Qiagen, Venlo, The Netherlands) DNA was modified by bisulfite treatment for the detection of methylated CpG residues, and was purified prior of the PCR amplification. Bisulfite-converted DNA was subjected to methylation-specific PCR (MSP) using primers specific for the hypermethylated form of glutathione-S-transpherase 1 (GSTP1) promoter (forward: 5'-GTTGCGCGGCGATTTC- 3'; reverse: 5'-GCCCCAATACTAAATCACGACG- 3' ). The PCR reaction was performed with 2.5 μl of bisulfite-modified DNA template in 25 μl of reaction mixture containing 2.5 μl 10 × PCR buffer, 200 μmol/L of each dNTP, 3.0 mmol/L MgCl2, 0.25 μmol/L each primer, 1.25 U of Ampli Taq Gold. The PCR reaction was subjected to hot start at 95°C for 10 minutes followed by 30 cycles of denaturation at 95°C for 30 seconds, annealing at 60°C for 1 minute, and extension at 72°C for 1 minute. A second set of primers detecting an unrelated not methylated gene, MYOD1 was used as control for the efficacy of bisulfite conversion (forward: 5'-CCAACTCCAAATCCCCTCTCTAT-3'; reverse: 5'-TGATTAATTTAGATTGGGTTTAGAGAAGGA-3'). Ten microL of each PCR reaction were loaded onto a 1.8% agarose gel containing ethidium bromide and PCR amplified DNA was visualized by UV transilluminator.

### Statistical analysis

SPSS for Windows (version 10.0.7) computer package was used for statistical analysis of the data. The study variables were normally distributed (Shapiro-Wilk test; P < 0.05). The Pearson's correlation test was used. We also evaluated the positive predictive value (PPV), the negative predictive value (NPV), the sensitivity and specificity of the performed method (biopsy print) with 95% Coefficient of Confidence (CI). Values < 0.01 were considered significant.

## Results

### Tissue print collection and print-phoresis from prostate biopsy

**Figure 1:**
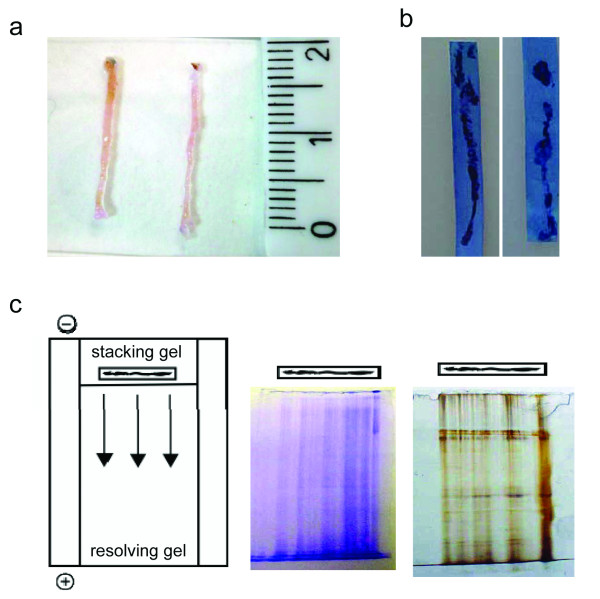
**Tissue print and print-phoresis**. (A) Photograph of two representative core needle biopsies. (B) The biopsy prints were incubated with 0.1% toluidine blue solution for five minutes. After two washing in phosphate buffer the prints were visualized as blue regions. (C) Schematic representation of the SDS-PAGE print-phoresis. Biopsy print has been put on the top of the resolving gel, and embedded in the stacking gel. Representative resolving gels were stained with Coomassie blue (left) or with silver solution (right) in order to visualize the protein content. On the top of each gel the position of the tissue print is shown.

We collected the tissue prints from the biopsies of prostate gland performed after RRP, TURP and TV adenomectomy. At least three different needle biopsies were performed in the tumour region by the same automatic biopsy gun used in routinely diagnostic procedures. The biopsy cores were about 15 mm in length and 1 mm wide (Figure 1a). Biopsy prints were obtained by spreading the biopsy cores on the nitrocellulose membrane. Once biopsy was removed, the presence of a cellular micropeel, copy of the biopsy surface, could be evidenced by toluidine staining (Figure 1b). Tissue print can be subjected to SDS-PAGE embedding it in the stacking gel (print-phoresis, Figure 1c). At the end of the electrophoretic run, gels were stained, according to standard procedures, with Coomassie blue or with silver staining (Figure 1c). Both staining procedures evidenced the presence of proteins migrated from the entire length of the tissue print.

### Zymogram analysis of biopsy print

**Figure 2:**
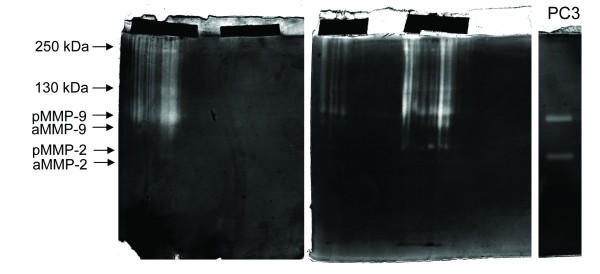
**Zymogram analysis of biopsy prints**. Two exemplificative gels, each one containing two tissue prints visible as dark strips in the stacking gel, are shown. The presence of discrete gelatinolytic activity is indicated on the left with the molecular weight or with the corresponding gelatinase. On the right the gelatinolytic pattern observed in conditioned medium from PC3 cells.

Biopsy prints were subjected to zymogram PAGE for the detection of gelatinolytic activity. Not all the biopsy prints demonstrated the presence of the lytic enzymes. When the gelatinolytic activity was evident we observed both a diffuse and a discrete lytic activity. The more frequent discrete bands were identified as MMP-2 and MMP-9 (pro- and active forms), as suggested by the correspondence with the gelatinolytic pattern by conditioned media from PC3 cells (Figure 2). Immunohistochemistry analyses of needle biopsies used for tissue prints were performed for verifying the presence of MMP-2 and MMP-9 and the results were expressed in a semiquantitative scale of intensity (BS, see material and methods). In order to analyze the relationship between gelatinolytic pattern in biopsy prints and the grading of prostate tumours we categorized the results obtained by zymogram analysis according to the following relative score (gelatinolytic score, GS): 0 for no gelatinolytic activity; 1 for diffuse gelatinolytic activity; 2 for discrete gelatinolytic activity (MMP-2 or MMP-9). Three different biopsy prints from the same patients were considered and the sum of GS and BS were calculated. According to this procedure we found a significant correlation between GS and BS (r = 0.97, p < 0.01) and between GS and the Gleason score (r = 0.86, p < 0.01), but not between GS and PSA value (Table 1). Also three biopsy prints from adenomas were analyzed by zymography, but in all cases no enzymatic activity was detected (Table 1).

**Table 1:** Gelatinolytic score calculated for biopsy prints of PCa and BPH biopsies

	Gleason Score	PSA (ng/ml)	Biopsy MMP9+MMP2 score (BS)	Gelatinolytic score (GS)	Correlation
**PCa**	9	7.0	5+2	4	BS vs GS = 0.97 * Gleason vs GS = 0.86 * PSA (PCa) vs GS = 0.02 PSA (all) vs GS = 0.36
**PCa**	8	6.0	5+1	4	
**PCa**	8	14.2	3+2	3	
**PCa**	7	8.3	3+0	2	
**PCa**	6	26.0	3+1	2	
**PCa**	6	8.2	2+0	0	
**PCa**	6	14.8	3+1	2	
**PCa**	6	5.9	0	0	
**PCa**	6	10.5	1+1	1	
**BPH**	--	3.0	0	0	
**BPH**	--	0.4	0	0	
**BPH**	--	3.2	0	0	

In the corresponding needle biopsies MMP2 and MMP9 expressions were evaluated by immunohistochemistry and a semiquantitative score (BS) was calculated. Correlation coefficients between BS and GS, between GS and Gleason score and between GS and PSA are shown. The correlation coefficient was calculated for all PSA values or only for PSA values from PCa patients (* *p*< 0.01)

### GSTP1 methylation analysis of biopsy print

**Figure 3:**
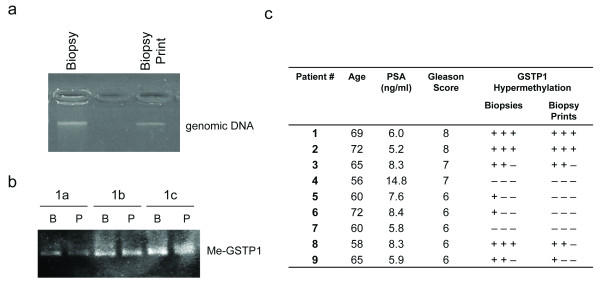
**DNA extraction from biopsy print and GSTP1 hypermethylation analysis**. (A) DNA was obtained from the biopsy print and from the corresponding biopsy and 1/5 of total DNA volume was subjected to electrophoresis run in 1.8% w/v agarose gel. (B) PCR-amplified products from bisulfite converted DNA were subjected to electrophoretic separation in agarose gel. DNA was extracted from three different biopsy prints (P) and the related biopsies (B) of the same patient (#1 as in 3C). (C) GSTP1 methylation status in PCa patients evaluated in three different biopsy prints and related biopsies (+ = GSTP1 hypermethylation).

Biopsy print can be used as source for DNA. At least 30 ng of genomic DNA from each biopsy print were recovered by extraction with proteinase K and its integrity and purity were comparable to DNA extracted by corresponding biopsy (Figure 3a). DNA was subjected to bisulfite conversion and clean-up and then amplified by PCR using specific primers for hypermethylated regions in the promoter of GSTP1 gene. The electrophoretic run of PCR products determined the presence of a unique amplified band that was of the same size in biopsies and in biopsy prints (Figure 3b). We analyzed for the presence of hypermethylated GSTP1 27 biopsies derived from 9 different patients and the corresponding 27 biopsy prints (Figure 3c). Moreover we analyzed 9 biopsies and corresponding biopsy prints from 3 different BPH, but all these samples resulted negative (data not shown). The concordance between biopsies and biopsy prints was of 85% (23/27). The discordances were observed only in 4 biopsy prints obtained by positive biopsies and that resulted negative. According to the data obtained, and considering BPH (6 patients) as negative controls, we compared the sensitivity, specificity, positive predictive value (PPV) and negative predictive value (NPV) of the biopsies and of the biopsy prints based on the three needle biopsies, performed on the area of the tumor, obtained for each of the 18 patients with an histopathological diagnosis of PCa after RRP (Table 2). These parameters were calculated considering positive those patients who had at least one biopsy (or biopsy print) positive for GSTP1 hypermethylation. The specificity of the two procedures resulted identical while the PPVs were comparable.

**Table 2:** Predictive parameters for PCa diagnosis associated to GSTP1 hypermethylation analysis in biopsy and in biopsy print

	Sensitivity % (95% CI)	Specificity % (95% CI)	PPV % (95% CI)	NPV % (95% CI)
**Biopsy**	77.8 (45.3-93.7)	50.0 (18.7-81.2)	70.0 (39.7-89.2)	60.0 (23.1-88.2)
**Biopsy print**	55.6 (26.7-81.1)	50.0 (18.7-81.2)	62.5 (30.6-86.3)	42.9 (15.8-75.0)

(CI = confidence interval)

## Discussion

Our study indicates that prostate core needle biopsy may be printed on a nitrocellulose membrane obtaining a cellular micropeel useful for several analytical procedures. Tissue print is easy and rapid to be performed without damaging the tissue, and it has been applied in numerous cellular models and with different experimental objectives.

Besides the technical application used, studies with tissue print can be divided into two categories: those focused on the spatial localization of the marker, and those looking for the presence of the marker. Tissue print can be utilized in hybridization assays or in histochemistry, detecting the position of the marker on it. Alternatively, the adhesive substrate can be used as source to extract proteins, mRNA or DNA. In this second circumstance, the tissue print has the main role of recovering a small portion of the tissue without affecting the integrity of the starting tissue. In our study we applied the second approach, and we demonstrated that biopsy print represents a suitable technique in recovering an amount of protein and DNA, sufficient to perform downstream analytical procedures. However, the zymogram print-phoresis allows also a spatial analysis of the specimen; in fact the core needle biopsy has an orientation "out-inside", containing prostatic tissue from the capsule toward the inner zone of the gland. For this reason, after marking the outer extremity of the biopsy, we may have a representation of the localization of gelatinolytic activity along the axis out-inside. In our preliminary study we frequently observed a non homogeneous MMPs localization in the biopsy print suggesting that a spatial differentiation in gelatinolytic activity could be possible.

Gelatin zymogram analysis was chosen because it is a relatively inexpensive technique that allows having unequivocal results. Moreover, this technique does not require the processing of the biopsy print, avoiding the loss or modification of the starting material. Moreover, it is well known that production of MMP-2 and MMP-9 in PCa cells is associated to tumor invasion and progression [3]. A significant correlation between the production of gelatinases and the Gleason score, but not between gelatinases and PSA values, was found. In order to limit the variability observed in the biopsy prints also from the same prostate gland, we analyzed at least three different biopsy prints. The highest gelatinolytic score assigned to the presence of discrete electrophoretic bands was always associated to specimens with a higher Gleason grade, while biopsy prints from BPH were always negative.

We performed the biopsy print in order to verify whether the DNA extracted from the prints was sufficient to detect the hypermethylation status of the GSTP1 promoter by methylation-specific PCR (MSP). The pattern of hypermethylation represents one of the most promising advancements in tumor diagnostic methods. Hypermethylation of CpG island sequences leads to gene silencing by preventing gene transcription and in human PCa cells, somatic GSTP1 CpG island hypermethylation and loss of GSTP1 expression appears to be the most common and consistent genome abnormality [10,11]. In our study, we obtained DNA amounts sufficient for MSP in all 27 PCa biopsy prints examined. We detected hypermethylation in GSTP1 promoter in 78% of patients (7/9), a value that is similar to those observed by other authors in PCa tissue [12]. The sensitivity of biopsy print-MSP resulted lower than those of biopsy-MSP (56% vs 79%). The decrease in sensitivity is due to a lower number of hypermethylation-positive biopsy prints respect to positive biopsies. This result should be probably accounted to the fact that the tissue print is a partial representation of the biopsy, and it collects DNA just from a limited portion of the tissue biopsy. For this reason if the printed tissue surface does not contain tumor cells or it contains tumor cells in a very low percentage, the tissue print may result negative also in presence of a positive biopsy. The decrease in sensitivity should be minimized by analyzing multiple biopsy prints from the same patient. On the contrary, we did not observe false positive in biopsy print samples, confirming the specificity of the biopsy analysis.

Both urologists and histopathologist point of view, who routinely work with us as a well integrated research group, underline that the future handling of patients with localized prostate cancer will undoubtedly depend upon a more sophisticated prognostication than that available today. The basis will continue to be the histopathological evaluation of tumor size, grade, localization and distribution within the gland unless the future need for objective techniques is well recognized. The option of combining an histopathological analysis of prostate biopsies to molecular information should be of importance in improving the current diagnostic procedures available, particularly in uncertain cases. Focusing on tissue print of prostate biopsies, the results of the current study are undoubtedly interesting unless need to be confirmed by the use in the clinical practise to verify whether it is really useful for the diagnosis of PCa. Because of widely adopted screening programs for the early detection of prostate cancer, several patients who undergo RP are diagnosed with tumors of small volume, and their extent and distribution in the gland can only be determined by a microscopic examination of the surgical specimen. Historically, one of the most important predictor of cancer control following RP is the absence of cancer at the surgical margins. Tissue print may be able in increasing specificity of the conventional histopathology mainly into the assessment of RP margins as it is a method for obtaining molecular information about the cancer that can add to the macroscopic and microscopic anatomical findings.

An important limitation of the present study is the low number of cases examined. However, this study has to be considered preliminary and performed in the attempt to check the methodology. In this regard we try to point out the promising aspects of the proposed methodology: first, biopsy print is a very simple and quick procedure that can be performed soon after the biopsy. The resulting print can be stored at -20°C until the execution of the analytic procedure, while the core needle biopsy can be processed for the histological analysis. Second, biopsy print resulted in an accurate copy of the needle biopsy offering reliable molecular information about the prostatic tissue. In conclusion, the application of biopsy print to a larger number of cases will clarify whether this promising technique should be useful in supporting the current procedures used for the diagnosis of PCa.

## Competing interests

The authors declare that they have no competing interests.

## Authors' contributions

AA carried out the methylation studies and drafted the manuscript. GP collected human specimens, performed the statistical analysis and helped to draft the manuscript. PS carried out the zymographies and prepared the figures. CV was the coordinator of clinical management of patients and participated in the design of the study. MB was the scientific coordinator and supervisor of the study and participated in its design. All authors read and approved the final manuscript.
